# Physiological and behavioral contagion/buffering effects of chronic unpredictable stress in a socially enriched environment: A preliminary study

**DOI:** 10.1016/j.ynstr.2024.100635

**Published:** 2024-04-11

**Authors:** Evren Eraslan, Magda J. Castelhano-Carlos, Liliana Amorim, Carina Soares-Cunha, Ana J. Rodrigues, Nuno Sousa

**Affiliations:** a Life and Health Sciences Research Institute (ICVS), School of Medicine, University of Minho, Braga, Portugal; b ICVS/3B's – PT Government Associate Laboratory, Guimarães, Portugal; c Faculty of Veterinary Medicine, Department of Physiology, Istanbul University-Cerrahpasa, Istanbul, Turkey; d P5 Clinical Digital Center, Braga, Portugal; e Clinical Academic Center (2CA), Braga, Portugal

**Keywords:** Stress buffering, Stress contagion, Rats, Enriched environment, Social behaviors, Oxytocin

## Abstract

Rodents are sensitive to the emotional state of conspecifics. While the presence of affiliative social partners mitigates the physiological response to stressors (buffering), the partners of stressed individuals show behavioral and endocrine changes indicating that stress parameters can be transmitted across the group members (contagion). In this study, we investigated the social contagion/buffering phenomena in behavior and neuroendocrine mechanisms after exposure to chronic stress, in groups of rats living in the PhenoWorld (PhW). Three groups were tested (8 stressed rats, 8 unstressed rats, and a mixed group with 4 and 4) and these were analyzed under 4 conditions: stressed (pure stress group, n = 8), unstressed (naive control group, n = 8), stressed from mixed group (stressed companion group, n = 8), unstressed from mixed group (unstressed companion group, n = 8. While naive control animals remained undisturbed, pure stress group animals were all exposed to stress. Half of the animals under the mixed-treatment condition were exposed to stress (stressed companion group) and cohabitated with their unstressed partners (unstressed companion group). We confirmed the well-established chronic unpredictable stress (CUS) effects in physiological, behavioral, and neuroendocrine endpoints; body weight gain, open arm entries and time in EPM, and oxytocin receptor expression levels in the amygdala decreased by stress exposure, whereas adrenal weight was increased by stress. Furthermore, we found that playing, rearing and solitary resting behaviors decreased, whereas huddling behavior increased by CUS. In addition, we detected significant increases (stress-buffering) in body weight gain and huddling behaviors between pure stress and stress companion animals, and significant stress contagion effects in emotional behavior and oxytocin receptor expression levels between naive control and control companion groups. Hence, we demonstrate buffering and contagion effects were evident in physiological parameters, emotional behaviors, and social home-cage behaviors of rats and we suggest a possible mediation of these effects by oxytocin neurotransmission. In conclusion, the results herein suggest that the stress status of animals living in the same housing environment influences the behavior of the group.

## 1 Introduction

Several studies show that rodents are sensitive to the emotional state of conspecifics (Karwicka et al., 2021; Meyza et al., 2017). Rats, in particular, were shown to sense the emotional state of other conspecifics and are capable of using socio-emotional cues to regulate their behavior (Andraka et al., 2021). Fear and pain transmission in rodents has been demonstrated in many studies (Meyza et al., 2017), and it has been reported that there is a bidirectional information transfer across rats in the context of potential danger (Han et al., 2019). When the presence of affiliative social partners mitigates the physiological response to stressors, this phenomenon is called social “buffering” (Kiyokawa and Hennessy, 2018). On the other hand, if the partners of stressed individuals show behavioral and endocrine changes indicating that stress parameters can be transmitted across the group members, this phenomenon is called “stress contagion/transmission” (Engert et al., 2019).

Recent studies have paid attention to stress contagion and buffering effects (Burkett et al., 2016; Carnevali et al., 2017; Sterley et al., 2018). It was previously reported that after foot-shock exposure for three weeks, the presence of an unstressed cage mate had revealed moderate stress-reducing effects, which were monitored in the open field test. Also, males housed with stressed females appeared to show signs of stress, had reduced growth rates, and exhibited a behavioral stress response, manifested by spending more time in the closed tube during the open field test (Westenbroek et al., 2005). In another study, unstressed prairie voles showed more consolidation behavior to foot-shock exposed stressed cage-mates, and unstressed animals' fear and anxiety-related behaviors and plasma corticosterone levels increased, indicating the stress contagion and empathy mechanism in rodents (Burkett et al., 2016). Furthermore, unstressed rats cohabitated with stressed partners exposed to four days of social defeat stress and showed social avoidance and fear in a new social context, with increased cardiac autonomic activation and hyperactivity of the HPA axis (Carnevali et al., 2017). Contagion of depression symptoms of chronic stress was also demonstrated in naive rats that cohabitated for five weeks with depressed-like rats (Boyko et al., 2015). Stress contagion in mice after foot-shock exposure, acute restraint-tail shock, and 14 days of restraint stress were also reported (Carneiro de Oliveira et al., 2017; Lee et al., 2021; Sterley et al., 2018). These findings suggest that the affective state of partners influence the behavioral and physiological responses of individuals in rodents.

The social environment is a prominent feature of daily life for species that live in groups. Although social interactions can change the effects of exogenous stressors, the majority of studies focused on dyadic encounters. To illustrate the importance of housing paradigms and consideration of variation in natural social behaviors visible borrow systems has been using that designed according to species ecology and mimic natural environments (Beery et al., 2020). Mainly, the potential effects of contagion/buffering effect of acute stress also have been tested between dyads housed in standard laboratory conditions. However, as stress buffering/contagion could occur on a larger scale, i.e., in group-living animals (Brandl et al., 2022), we thought of interest to investigate the social stress contagion/buffering phenomena and related neuroendocrine mechanisms after exposure to chronic stress, in larger groups of rats living altogether in a natural enriched environment. Since PhW stimulates positive affective states, enables the establishment of social interactions during the active period, and provides rats to perform more species-specific behaviors (Castelhano-Carlos et al., 2014, 2017), investigating contagion/buffering phenomenon in such a context would increase the quality and validity of these concepts.

## 2 Material methods

### 2.1 Animals and experimental design

**Fig. 1:**
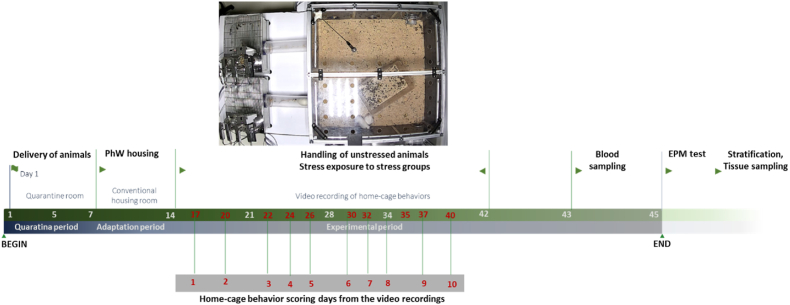
A photo of PhenoWorld and experimental procedures. 7–8 weeks old of Wistar rats were held in quarantine for 7 days, afterwards moved to conventional housing room. After 7 days of adaptation period here, unstressed animals were only handled, stressed groups were exposed to chronic unpredictable stress for 4 weeks. Home-cage behaviors were recorded for total of 10 days during the experimental period. After this period, blood samples were obtained for corticosterone analyses. EPM test was conducted, the animals were killed at the end of the experiment and tissue samples were obtained.

Wistar Han male rats, aged 7–8 weeks, which were purchased from Charles River Laboratories (Saint Germain Nuelles, France), were used in this study. Upon arrival, animals were placed and kept in a quarantine room for one week. Afterwards, they were transferred to a standard housing room and housed under standard laboratory conditions (artificial 12/12 light-dark cycle, lights on from 8:00 a.m. to 8:00 p.m., relative humidity of 50–60 % and 22 °C ambient temperature) with *ad libitum* access to food (4RF21, Mucedola SRL, Settimo Milanese, Italy) and water (autoclaved tap water). A total of 8 animals in each experimental condition were placed in the PhW (TSE Systems GmbH, Bad Homburg, Germany). PhW is a standard filter topped transparent cage of 610 × 435 × 215 mm, with 2065 cm^2^ floor area (ref. 2000P, Tecniplast, Buguggiate, Italy) that allows the study of complex group behaviors (Amorim et al., 2022; Leite-Almeida et al., 2022). The PhW setup consists of a 1 m^2^ area and a 50 cm high central cage with corncob bedding on the floor. The central cage is connected to two drinking/feeding boxes through two open-access tubes. All areas were covered either by perforated Plexiglas or stainless-steel grids. Cardboard tubes were provided to all groups as environmental refinement, and a standard type III cage were placed in the central area of the PhW for jumping and climbing (Castelhano-Carlos et al., 2014). All groups had an adaptation period of one week in the conventional housing room before initiating the experiments. Four independent conditions were analyzed: one in the control condition (naive control group, n = 8), one in the pure stress condition (pure stress group, n = 8), and two in the mixed-treatment conditions (unstressed companion, n = 8 and stressed companion groups, n = 8). All groups were formed by joining four groups of 2 animals living previously in different standard cages. Naive control animals remained undisturbed except for cage cleaning and daily handling procedures. Pure stress group animals were all exposed to stress. These two groups were also so-called same-treatment groups. Half of the animals under the mixed-treatment condition was exposed to stress (stressed companion group) and cohabitated with their unstressed partners (unstressed companion group). The same experimenter handled the animals during the study. All experimental procedures are shown in Fig. 1.

All experiments were carried out in accordance with the European Directive 2010/63/EU and the Portuguese regulations and laws (Decreto-Lei 113/2013 and Decreto-Lei 1/2019) on the protection of animals used for scientific purposes of the Ministry for Agriculture, Ocean, Environment and Spatial Planning, which authorized the project in which this study was included (DGAV authorization code 9458). The animal study was reviewed and approved by the ethics committee of the University of Minho, and authorized by the Portuguese national competent authority.

### 2.2 Chronic unpredictable stress (CUS)

We used chronic unpredictable stress (CUS) paradigm which is a validated stress protocol that was previously described and proven to induce physiological and behavioral alterations typical of the chronic stress response in previous studies and also has been successfully utilized in our laboratory (Eraslan et al., 2023; Magalhães et al., 2017; Ventura-Silva et al., 2020). One of several stressors was applied in random order and at different intervals of the light phase of the day, daily for four sequential weeks. During the stress exposure animals were randomly separated in groups depending on the type of the stressor applied. Stressors were applied in a separate experimental room from where the animals were housed, while unstressed group animals were handled for the same time. The stressors were: exposure to cold water (18 °C), overcrowding, vibration, restricted space, and a hot air stream as detailed in Table 1.

**Table 1:** Chronic unpredictable stress paradigm.

Overcrowding	A total of 8 animals were placed in STD6 cages (610 × 435 × 215 mm) for 1 h
Restricted space	Four animals were confined in per STD2 cages (425 × 266 × 185 mm) for 1 h
Exposure to the hot air stream	Animals were exposed to a hot air stream ranging from 45 to 50 °C for 45 min. They were placed in STD cages (610 × 435 × 215 mm) in groups of four.
Cold water	Replacement of bedding material with cold water, 400 ml (18 C) for 1 h. They were placed in STD cages (610 × 435 × 215 mm) in groups of four.
Vibration	Placement on a vibrating/rocking platform for 15 min.

Weekly body weights and post-mortem adrenal weights were recorded to evaluate the impact of stress exposure.

### 2.3 Serum corticosterone

At the end of the experiment, blood samples were collected for corticosterone assessment. Collection was performed at two different time points; within 1 h after lights on and after lights off by a tiny incision on a dorsal tail-vein. After collection, blood samples were centrifuged at 16000 × G for 10 min (Biofuge Fresco, Heraeus, Osterode, Germany). Serum was removed and stored at – 80 °C until further analyses.

Corticosterone levels were measured by the enzyme-linked immunosorbent assays (ELISA) using a commercial kit according to the manufacturer's instructions (ADI-900-097, Enzo Life Sciences, Lausen, Switzerland). The absorbance at 405 nm was measured using a microplate reader. The minimum detectable level of corticosterone concentration was 26.99 pg/ml. The average intra-assay coefficient of variation (CV) measured was of 2.4%. The average inter-assay CV were 7 and 5 % for nadir and peak corticosterone levels.

### 2.4 Elevated plus maze

Elevated plus maze (EPM) was used for assessing the emotional behaviors of animals. The test apparatus (ENV-560; Med Associates Inc., St. Albans, VT, USA) was a black polypropylene plus-shaped platform with two open (50.8 × 10.2 cm) and two closed (50.8 × 10.2 × 40.6 cm) arms, heightened 72.4 cm above the floor. The junction area between the four arms measured 10 × 10 cm. The experimental room was lit by 40 W fluorescent lamps mounted above the maze so that all arms were equally illuminated (300 lx at the maze floor level). To record the behaviors, a charge-coupled device (CCD) camera was placed above the maze. Animals were tested for 5 min. They were placed in the center of the apparatus facing one of the open arms at the beginning of the test. To eliminate any odor cues, the maze was cleaned using ethanol solution (70%) and wiped dry between trials. Time spent in closed and open arms and the number of entries into each arm of the maze were obtained by behavioral observation of recorded video tapes.

The percentages of time spent in the open arms (100 × time spent in the open arms/total time spent in the open and closed arms) and also the percentage frequency of entries in the open arms (100 × number of entries into open arms/total entries into all arms), and total arm entries (total number of closed and open arm entries) were calculated as an index of emotional behavior.

### 2.5 Observation of home-cage behaviors

The home-cage behaviors of rats were recorded by surveillance video cameras installed above PhW. Measuring behavior over a limited time at present intervals, time-sampling model, was used for the scoring (McCormick et al., 2007; Saibaba et al., 1996). An arbitrary time window of 1-h periods was chosen in each observation day within the first 2 h of the dark phase for a total of 10 different observation periods during the experimental period (days 14–42). Behaviors of each animal were scored as frequencies within the first 5 min of every 10-min interval for 1 h (5 × 6 = 30 min) performed for 10 days (300 min). The results monitored across a total of ten days were pooled into a single value (mean).

The observed behaviors were classified into two categories: social activities, such as social play, social investigation, allogrooming, huddling, and following, and non-social activities, such as self-grooming, walking, solitary resting, rearing, and digging (Cirulli et al., 1996; Draper, 1967; Niesink and Van Ree, 1982; Saibaba et al., 1996; Vanderschuren et al., 1997) (Table 2).

**Table 2:** Ethograms of rat behavior in the home-cage.

Behavioral Element	Description
Social play	One animal approach and soliciting another (pouncing; attempt to nose or rub of the neck of the partner), chasing the partner, crawling over/under, boxing, wrestling, pinning (lying with the dorsal body surface on the floor with the other animal standing over it), and lateral display
Social investigation (sniffing)	Sniffing of the any part of the body of the cage-partner
Allogrooming	The grooming of one animal by another
Huddling	The presence of at least 2 animals, resting or sleeping while maintaining close physical contact with the partner
Following	Walking or running in the direction of the partner moving away

Self-grooming	Repeatedly cleaning the body fur using forelimbs beginning with the snout, progressing to the ears and ending with whole body grooming
Walking	Leg movement enabling the animal's slow pace across the floor
Solitary resting	Immobile and not in contact with cage partner
Rearing	Standing on the hind paws and stretching the body while sniffing the air from the upper part of the cage
Digging	Pushing, pulling or kicking bedding material away and around using the snout and/or both the forepaws and hind paws

All videos of behaviors were scored by the same observer and the second observer scored part of the videos to control for a possible bias.

### 2.6 Post-mortem verifications

All rats were killed by decapitation under intraperitoneal sodium pentobarbital (20% Eutasil®, Sanofi, Gentilly, France) anesthesia at the end of the study. After decapitation, the brain was rapidly removed and placed on an ice-chilled Petri plate. The whole amygdala was dissected from the brains (Paxinos and Watson, 2007) and stored at – 80 °C until further analyses. A necropsy was performed, and the adrenal glands were taken out, cleaned out of the surrounding tissues, and weighed (PR503, Mettler Toledo). The weight of the adrenal glands was divided by the weight of the rat to assess “relative weight,” and these data were used for later analyses.

### 2.7 RNA isolation and quantitative real-time PCR

Brain samples were initially homogenized in an ice-cold Trizol (Invitrogen, Carlsbad, CA). Then, total RNAs were extracted from amygdala using Trizol, according to the manufacturer's instructions. Afterwards, the RNA samples were measured and evaluated by a NanoDrop spectrophotometer, and trace DNA contamination was removed by DNase digestion. cDNA was synthesized from 1 μg total RNA using the iScript cDNA Synthesis kit (Bio-Rad). Quantitative real-time PCR analyses were performed using SsoFast EvaGreen Supermix (Bio-Rad) with a CFX96 Real-Time PCR Detection System (Bio-Rad) to measure the rats' OXTR mRNA levels. The following primers were designed to amplify rat OXTR (Fw, CAAGGAAGCTTCTGCCTTCATCATT; Rw, CACGAGTTCGTGGAAGAGGT), and rat Beta-2-Microglobulin (Fw, GTGCTTGCCATTCAGAAAACTCC; Rw, AGGTGGGTGGAACTGAGACA). Expression levels of housekeeping gene rat Beta-2 Microglobulin (B2m) were used for normalization. The results are presented as relative expression to the housekeeping gene (ΔCt).

### 2.8 Statistics

Data were initially evaluated for normality using the Shapiro-Wilk test. Since the data was normally distributed, parametric tests were used. Outliers were excluded using the ROUT method (Motulsky and Brown, 2006). Body weight gains were analyzed by 2 × 2 repeated measures analyses of variances (ANOVA). The main effects and interactions were evaluated, and simple main effects were analyzed when interactions were present. Two different time points (nadir vs. peak) of corticosterone levels were analyzed by repeated measures ANOVA. A two-way (2x2) between-subjects ANOVA was conducted to compare the main effects of treatments and the interaction effects on adrenal weights, each of the sampling points of corticosterone measurements, EPM, NSF, home-cage behaviors, and gene expression results. Simple main effects were analyzed when interaction was determined between the factors.

The level of statistical significance was set to *p* ≤ 0.05. All analyses were performed using SPSS software version 21 (SPSS Inc., Chicago, IL, USA).

## 3 Results

### 3.1 Body weight gain (%)

**Fig. 2:**
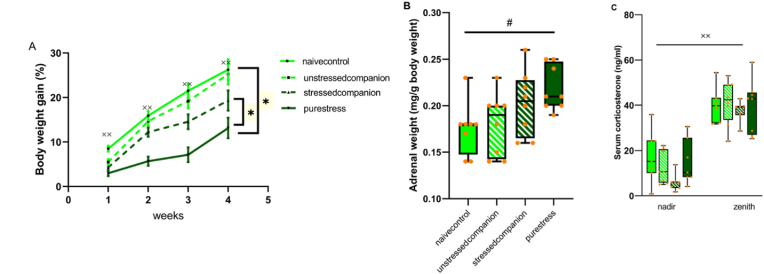
Biometric data **A)** Body weight gain (%) of animals during the experiment. Data is presented as means ± s.e.m **B)** The relative adrenal weights of animals (mg/g body weight) **C)** Serum corticosterone levels (ng/ml) at the end of the experimental period at nadir (8–9 a.m.) and zenith (8–9 p.m.). Data is presented by box plots where the central lines represent the median, and the whiskers represent the minimum and maximum values **(B, C).**^xx^*p*<0.001 and ^#^*p* < 0.05 indicating the general effect of time and stress, respectively.

A comparison regarding this parameter was done, to search for a possible “companion buffering/contagion” effect (in simple terms, we wanted to know whether control animals would trigger a beneficial influence on stressed animals or if stressed animals would produce a detrimental effect on control animals). First, we confirmed that there was a significant effect of time, with all animals increasing body weight throughout the experiment (*F* (3, 84) = 200.34, *p* < 0.001). We also observed a significant stress x companion-treatment interaction (*F* (1, 28) = 7.66, *p* = 0.01), as pure stress animals displayed lower body weight gain than naive control animals (*F* (1, 14) = 103.27, *p* < 0.001). In addition, we also observed a beneficial influence of the social companion in stressed animals as the body weight gain was higher in stressed animals living with unstressed than in those living solely with stressed animals (*F* (1, 14) = 13.31, *p* = 0.003) (Fig. 2A).

### 3.2 Adrenal weights

There was a significant main effect of stress on adrenal weights (*F* (1,28) = 9.44, *p* = 0.005), where stressed animals adrenal weights were higher than those of unstressed animals (Fig. 2B).

### 3.3 Corticosterone levels

We determined the corticosterone levels at the nadir and zenith and found that corticosterone levels of animals were lower when the lights were on (8–9 h) and higher with the lights off (20–21 h) in all conditions (*F* (1,27) = 189.83, *p* < 0.001) (Fig. 2C).

### 3.4 Emotional behavior

**Fig. 3:**
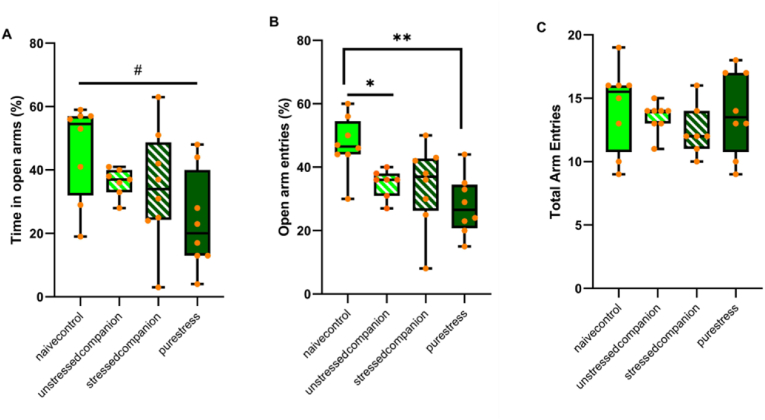
Behavioral data obtained in the EPM **A)** The percentage of time spent in the open arms **B)** The percentage of open arm entries **C)** Total arm entries of animals. Data is presented by box plots where the central lines represent the median, and the whiskers represent the minimum and maximum values.; ^#^*p* < 0.05, *p < 0.05, and **p < 0.001 indicating the general effect of stress exposure and stress x companion-treatment interactions, respectively.

Stress exposure affected the percentage of time spent in the open arm in the EPM. Stressed animals spent less time in the open arms of the maze than unstressed animals (*F* (1,27) = 5.25, *p* = 0.03). Furthermore, there was a tendency for the stress and companion-treatment interaction to be significant on this parameter (*F* 1,27) = 3.87, *p* = 0.06). Naive control animals' time scores in open arms were higher than those of pure stress animals (*F* (1,14) = 8.63, *p* = 0.01), while the difference between mixed-treatment groups was not significant (Fig. 3A).

Stress x companion-treatment interaction was significant on emotional behavior when considering the ratio of open/total arm entries (*F* (1,27) = 7.22, *p* = 0.01) in the EPM. Stress exposure significantly affected same-treatment group animals’ behavior. Pure stress animals had lower entries (*F* (1,14) = 17.52, *p* = 0.001) than naive controls. In addition, in the companion treatment condition, unstressed animals living with stressed animals had lower entries (*F* (1,13) = 10.95, *p* = 0.006) than those of naive controls (Fig. 3B).

Neither stress nor companion-treatment had a significant effect on total arm entries (Fig. 3C).

### 3.5 Home-cage behavior

#### 3.5.1 Social activities

**Fig. 4:**
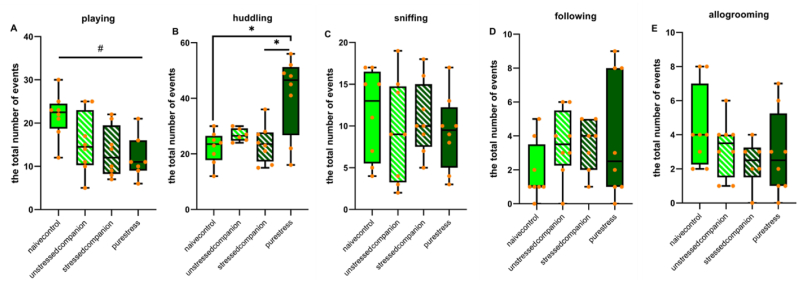
Analyses of social activities in home-cage behaviors (the total number of events). Social play, huddling, sniffing, following, and allogrooming behaviors. Data is presented by box plots where the central lines represent the median, and the whiskers represent the minimum and maximum values.; ^#^*p* < 0.05 and *p < 0.05, indicating the general effect of stress exposure and stress x companion-treatment interactions, respectively.

Stress had a significant effect on playing where unstressed animals had higher scores than stressed animals (*F* (1,27) = 7.82, *p* = 0.009; Fig. 4A).

The interaction between stress and companion-treatment was significant in huddling behaviors (*F* (1,26) = 11.33, *p* = 0.02; Fig. 3B). Naive controls huddling behavior was lower than pure stress animals (*F* (1,14) = 11.79, *p* = 0.004; Fig. 4B). Considering companion-treatment condition, stressed companions had lower scores of huddling compared to pure stress animals (*F* (1,14) = 9.47, *p* = 0.008).

Stress and companion-treatment conditions did not affect sniffing, following and allogrooming behaviors (Fig. 4C–E).

#### 3.5.2 Non-social activities

**Fig. 5:**
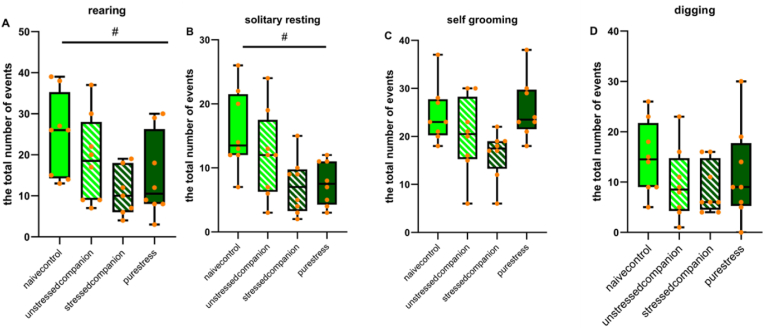
Analyses of non-social activities in home-cage behaviors (the total number of events). Rearing, solitary resting, self-grooming, and digging behaviors. Data is presented by box plots where the central lines represent the median, the whiskers represent the minimum and maximum values.; ^#^*p* < 0.05 indicating the general effect of stress exposure.

We observed a significant effect of stress regarding rearing (*F* (1,27) = 6.98, *p* = 0.01; Fig. 5A) and solitary resting behaviors (*F* (1,28) = 11.37, *p* = 0.02; Fig. 5B) where unstressed animals had higher scores than those of stressed animals.

Self-grooming and digging behaviors did not change by the treatments (Fig. 5C and D).

### 3.6 Amygdala oxytocin receptor mRNA expression

**Fig. 6:**
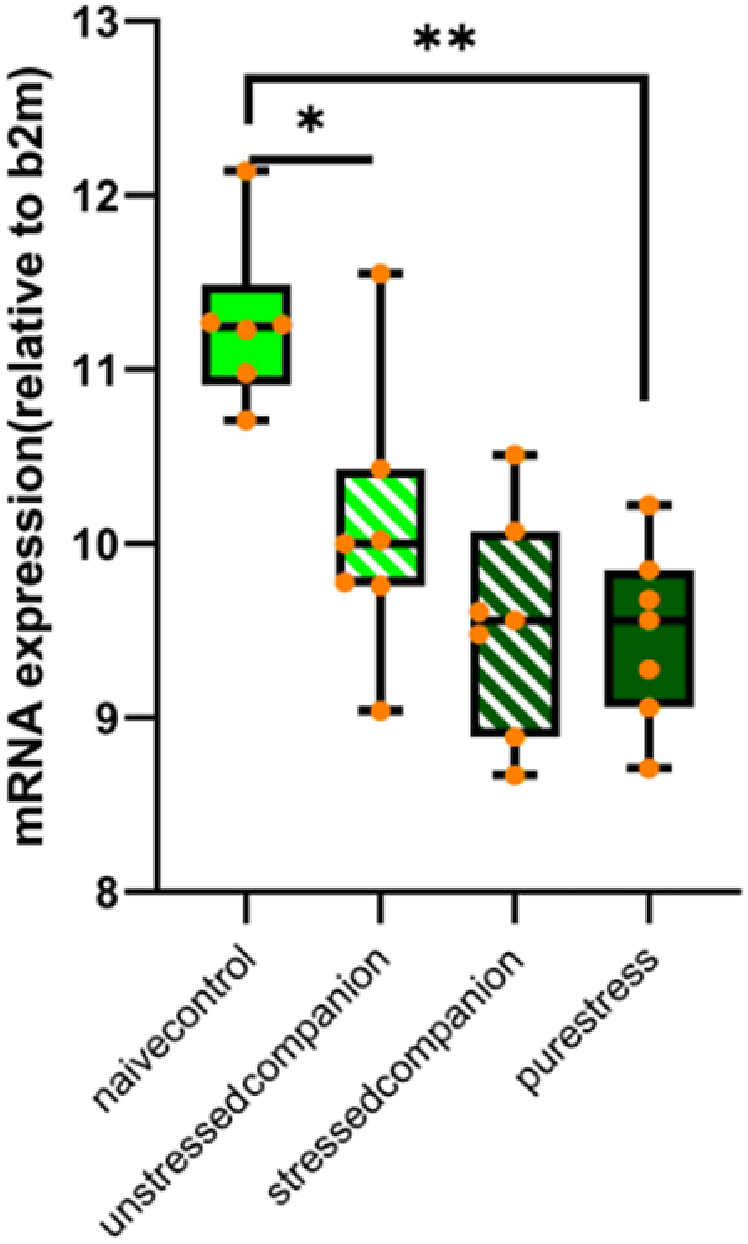
Relative mRNA expression levels of oxytocin receptors in the amygdala regarding dCt values normalized to housekeeping gene B2M. Data is presented by box plots where the central lines represent the median, and the whiskers represent the minimum and maximum values.; *p < 0.05, and **p < 0.001 indicating the stress x companion-treatment interactions.

There was a significant interaction between stress and companion-treatment on amygdala oxytocin mRNA expression (*F* (1,23) = 6.89, *p* = 0.02). Stress exposure revealed a significant effect on both same-treatment conditions. Oxytocin receptor mRNA levels of pure stress animals were lower than naïve controls (*F* (1,11) = 41.99, *p* < 0.001) (*F*(1,16) = 11.04, *p* = 0.011). In addition, unstressed companion animals’ oxytocin expression was lower than naïve controls regarding unstressed groups (*F*(1,11) = 10.51, *p* = 0.008) (Fig. 6).

## 4 Discussion

The main objective of our study was to investigate the physiological and behavioral contagion/buffering effects of chronic unpredictable stress (CUS) on rats including the procedure of social housing in rats living in a socially enriched environment (Phenoworld, PhW). We, therefore, tested whether cohabitation with a CUS exposed partners/group or vice versa cohabitation with unstressed partners/group would have elicited physiological and behavioral changes in the cage-mates in the PhW. Herein we demonstrated that CUS resulted in physiological and behavioral changes, some of which are specific to companion-treatment conditions. We detected stress contagion and buffering effects among cage-mates indexed by changes in investigated parameters obtained from rats that cohabitated in groups with stressed/unstressed partners.

Regarding physiological parameters, BWG, and adrenal weights, we found that the effect of stress exposure was evident. While the adrenal weight of stressed animals was higher than unstressed groups, the effect of stress on BWG was apparent in same-treatment group animals; body weight gain was lower in pure stress animals than in naive controls, reflecting the impact of the stress exposure (Cullinan and Wolfe, 2000; Eraslan et al., 2023; Sousa et al., 1998). However, we did not observe this effect in companion-housing conditions suggesting living with stressed/unstressed partners changes the physiological response of animals. Considering the companion-treatment effect we found evidence for stress buffering effect on BWG of animals exposed to chronic stress.

In terms of emotional behavior, known to be affected by stress exposure (Jacinto et al., 2017; Ventura-Silva et al., 2013), we confirmed the adverse effect of CUS in the EPM as pure stress animals tended to spend less time in the open arms of the EPM and also had significantly fewer entries into open arms in EPM compared to naive control groups. Noticeably, in this behavioral domain, we found a clear stress contagion effect. We observed that unstressed companion animals entered less in open arms than naïve controls in EPM indicating contagion of stress (and there was a similar tendency for time spent in the open arms). Such observation is in line with a previous study in which 14 days of restraint stress was reported to induce anxiogenic behaviors in the unstressed cage mates of stressed companions (Carneiro de Oliveira et al., 2017).

We next examined home-cage behaviors of animals to assess the presence of social stress contagion/buffering effect. In line with previous studies, herein we showed that stressed animals' playing, rearing, and solitary resting activities were lower, and huddling behaviors were higher than unstressed animals indicating the detrimental effect of stress on home-cage behavior of rats (Babb et al., 2014; Eraslan et al., 2023; Saxena et al., 2021). Of interest, the increase in huddling behavior observed after stress exposure can be interpreted as a potential sign of greater affiliation and social bonding (Crockford et al., 2017; Muroy et al., 2016), and a strategy to cope with the negative effects of stress exposure. These changes we observed in social and non-social behaviors of rats exposed to stress are in accordance with the results of our previous study (Eraslan et al., 2023). Interestingly, in addition, we found evidence for the buffering effect of stress in huddling behaviors, as stressed companion animals had lower huddling behavior than those of pure stress animals.

Lastly, in a preliminary exploration of a possible explanation for these behavioral findings, we measured the OXRR levels in the amygdala. OXT is known to have stress-protective and anxiolytic effects (Neumann and Slattery, 2016; Ring et al., 2006) and low levels of OXTR expression in brain regions responsible for emotional and social behaviors are hypothesized to be associated with high anxiety levels (Neumann and Slattery, 2016). Variations in maternal care behaviors of rats are also associated with differences in oxytocin receptor expression levels in brain regions that are known to mediate the expression of maternal care in rats (Francis et al., 2000). Acute peripheral injection of oxytocin was reported to have revealed an increase in the pro-social behavior of adjacent lying in rats (Ramos et al., 2013). A positive correlation was demonstrated between medial amygdala OXTR binding and social investigation scores in male rats (Dumais et al., 2013). Furthermore, a positive correlation between social interaction and medial amygdala, hypothalamic PVN OXTR expression in male mice was documented, indicating the role of OXTR in mediating social interactions (Murakami et al., 2011). Stress-related downregulation of amygdala OXTR and the decrease in pro-social behaviors after a single prolonged stress application restored after the intranasal OXT application also show the role of oxytocin in stress-related sociability (Wang et al., 2019; Muroy et al., 2016) reported that acute immobilization stress increased social support-seeking behavior and hypothalamic oxytocin signaling in male rats (Muroy et al., 2016). However, all these effects of oxytocin on prosocial behaviors were investigated under acute stress or neutral conditions; there is only a single study showing increased OXTR expression in amygdala chronic stress in female rats (Nowacka-Chmielewska et al., 2017). In contrast, we found a decrease in the OXT receptor levels in the amygdala in the chronically stressed animals. Interestingly, the OXTR mRNA levels of unstressed companion animals were also lower than those of naive control animals. In a mice study, the oxytocinergic system in the central amygdala was shown to have been involved in emotional recognition; and discrimination of positive and negative emotional states in conspecifics (Ferretti et al., 2019), which is the initial step of emotional contagion. Therefore, considering the higher OXTR mRNA levels observed in the naive control group when compared to the unstressed companion group it is tempting to establish a putative mechanistic link between OXT neurotransmission and stress contagion.

This study presents several limitations. We may have failed to capture the dynamic nature of HPA axis shift in response to stress and social environments by only conducting end-point hormone sampling; part of this limitation is mitigated by other determinations of neuroendocrine status. We have only explored the role of OXT neurotransmission in the context of the present study and only in one brain region (without dissecting the difference between the sub-divisions of the amygdala); in addition, we detected OXT receptor mRNA levels but not protein levels and it is known that differences of mRNA do not always translate to differences in proteins. However, this represents an exploratory approach for a mediation effect and OXT is reported to be relevant to the context of this study. Lastly, in the experimental design we have used, there are no animals coming from multiple cohorts of replicate groups e, and in light of the high variation of behavioral data, the statistical power is low; however, it should be highlighted that the results herein reported in the comparison between controls and stressed rats are in line with previous published results (Eraslan et al., 2023). The most of the stress buffering/contagion studies investigated this phenomenon on only male rodents (Bowen et al., 2013; Carneiro de Oliveira et al., 2017; Karwicka et al., 2021; Kiyokawa et al., 2016, 2018; Nakamura et al., 2016; Nakayasu and Kato, 2011) and just a few studies both male and females were used (Insana and Wilson, 2008; Sterley et al., 2018). To compare the sex differences, a future study should be planned examining buffering/contagion effects on both males and females.

In conclusion, the results herein present suggest that the stress status of animals living in the same housing environment influences the behavior of the group. Whether these results can transfer to other species, including humans needs to be explored but understanding the neurobiological processes underlying stress contagion/buffering effects among groups seems of relevance for mental and physiological health.

## Funding

This work has been funded by 10.13039/501100005635Fundação Calouste Gulbenkian [contract grant P-139977]. Part of this work was funded by 10.13039/501100019370Foundation for Science and Technology (FCT) by National funds, through projects UIDB/50026/2020 and UIDP/50026/2020, and by project PTDC/MED-NEU/29071/2017 (REWSTRESS).

## Unlisted references

Paxinos and Watson, 2007

## CRediT authorship contribution statement

**Evren Eraslan:** Writing – review & editing, Writing – original draft, Methodology, Investigation, Formal analysis, Data curation, Conceptualization. **Magda J. Castelhano-Carlos:** Writing – review & editing, Methodology, Investigation, Data curation. **Liliana Amorim:** Writing – review & editing, Methodology, Investigation, Data curation. **Carina Soares-Cunha:** Writing – review & editing, Methodology, Investigation, Formal analysis, Data curation. **Ana J. Rodrigues:** Writing – review & editing, Supervision, Methodology, Investigation, Conceptualization. **Nuno Sousa:** Writing – review & editing, Writing – original draft, Validation, Supervision, Methodology, Investigation, Funding acquisition, Formal analysis, Conceptualization.

## Declaration of competing interest

The authors declare that they have no known competing financial interests or personal relationships that could have appeared to influence the work reported in this paper.

## Data Availability

Data will be made available on request.
